# Analysis of the association between spawning time QTL markers and the biannual spawning behavior in rainbow trout (*Oncorhynchus mykiss*)

**DOI:** 10.1590/S1415-47572010000300032

**Published:** 2010-09-01

**Authors:** Nelson Colihueque, Rosy Cárdenas, Lorena Ramírez, Francisco Estay, Cristian Araneda

**Affiliations:** 1 Departamento de Ciencias Básicas, Universidad de Los Lagos, OsornoChile; 2 Piscicola Huililco Ltda., Centro Ojos del Caburgua, PucónChile; 3 Departamento de Producción Animal, Facultad de Ciencias Agronómicas, Universidad de Chile, Santiago de ChileChile

**Keywords:** association analysis, biannual spawning, microsatellite markers, rainbow trout

## Abstract

The rainbow trout is a salmonid fish that occasionally exhibits broodstocks with biannual spawning behavior, a phenomenon known as a double annual reproductive cycle (DARC). Spawning time quantitative trait loci (SPT-QTLs) affect the time of the year that female rainbow trout spawn and may influence expression of the DARC trait. In this study, microsatellite markers linked and unlinked to SPT-QTLs were genotyped to investigate the underlying genetics of this trait. SPT-QTLs influenced the DARC trait since in two case-control comparisons three linked markers (*OmyFGT12TUF*, *One3ASC* and *One19ASC*) had significant levels of allelic frequency differentiation and marker-character association. Furthermore, alleles of *One3ASC* and *One19ASC* had significantly higher frequencies in populations that carried the DARC trait.

Some rainbow trout (*Oncorhynchus mykiss*) broodstocks spawn twice a year, an unusual phenomenon known as the double annual reproductive cycle (DARC) or biannual spawning behavior (Hume, 1955; Aida *et al.*, 1984; Gall and Crandell, 1992). The two spawnings occur at regular intervals of approximately six months: the first during a normal reproductive cycle and the second during an additional reproductive cycle. Only a fraction of the females that spawn during the normal cycle experience a second spawning (Aida *et al.*, 1984). Broodstocks that carry the DARC trait have been the subject of various reproductive studies (Aida *et al.*, 1984; Lou *et al.* 1984; Tazaki *et al.*, 1993; Takano *et al.*, 1995), although the underlying genetics of this trait remain largely unknown. Another reproductive trait possibly related to DARC in rainbow trout is known as spawning time (SPT) (Siitonen and Gall, 1989). This trait influences the time of year that females spawn and is controlled by numerous quantitative trait loci (QTLs) (Sakamoto *et al.*, 1999; Fishback *et al.*, 2000; O'Malley *et al.*, 2003). Several markers closely linked to these chromosomal segments have been described. We propose that the underlying genetics of the DARC character in rainbow trout is similar to that of the SPT trait since both are related to the time of year when breeders spawn. To test this hypothesis, we undertook a marker-character association analysis for the DARC trait based on a panel of microsatellite markers closely linked to SPT-QTLs in rainbow trout.

Two broodstocks, Wytheville 02 (Wt-02, n = 52) and Wytheville 05 (Wt-05, n = 28) with a DARC trait frequency of 14%-35%, were used. The control stock, Steelhead (Sh, n = 35), had no DARC trait. These broodstocks were obtained from Piscicola Huililco Ltda., a commercial fish hatchery in southern Chile (39°28'04” S, 71°49'56” W). The DARC character was detected in this hatchery in 2001 in specimens that displayed this trait spontaneously. In these individuals, the DARC trait was characterized by a normal reproductive cycle (March-July; spring spawning) and an additional reproductive cycle (September-December; spring spawning). Blood samples were collected from a caudal vein and DNA was extracted by the phenol-chloroform method, as previously described (Taggart *et al.*, 1992).

Five microsatellite markers linked to SPT-QTLs (*OmyFGT12TUF*, *One3ASC*, *One19ASC*, *One112ADFG* and *Ssa103NVH*) and four microsatellite markers not linked to these chromosomal regions (*OmyFGT14TUF*, *OmyFGT15TUF*, *Omy27DU*, *Omy207UoG*) were used (Table 1). The selected linked markers belonged to three different linkage groups in which a strong effect of QTLs on the SPT trait has been observed with significant association (p < 0.05) (Sakamoto *et al.*, 1999; Fishback *et al.*, 2000; O'Malley *et al.*, 2003): *One19ASC* in linkage group OA-XXIV, *One3ASC* and *Ssa103NVH* in linkage group OA-XIX and *One112ADFG* in linkage group OA-VIII (Nichols *et al.*, 2003) (Figure 1). The selected unlinked markers belonged to linkage groups that were different from those of the selected linked markers (*OmyFGT14TUF* in linkage group OA-X, *Omy27DU* in linkage group OA-II and *Omy207UoG* in linkage group OA-VIII) (Sakamoto *et al.*, 1999, 2000; O'Malley *et al.*, 2003) in which no association with SPT-QTL has been reported (Sakamoto *et al.*, 1999; O'Malley *et al.*, 2000). *OmyFGT15TU*F was considered to be unlinked since although it maps in the linkage group OA-III where a SPT-QTL exists (Sakamoto *et al.*, 1999) there was no significant association with this QTL. The microsatellite markers were genotyped by electrophoresis in 6% polyacrylamide 7 M urea gels after amplification by PCR. The PCR mix consisted of 1 x *Taq* polymerase buffer, 0.13-0.28 μM of dNTPs, 1.3-2.5 mM MgCl_2_, 0.26-0.4 μM of each primer, 0.02 U of *Taq* polymerase/μL (Invitrogen) and 40 ng of template DNA/μL in a final volume of 15 μL. Amplicon size was determined by using a 25 bp DNA standard. The thermal profiles were standardized for each microsatellite based on the annealing temperature of the corresponding primer pair.

**Figure 1:**
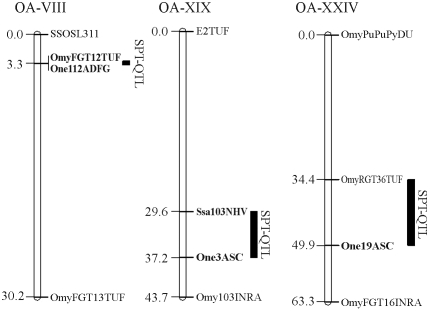
Map positions of the markers linked to spawning time QTLs used in this work (indicated in bold). The map distance (in centiMorgans) between adjacent markers is shown on the left. The locations of the spawning time QTLs (SPT-QTLs) are indicated by solid bars. Each linkage group was defined as proposed by Nichols *et al.* (2003). Linkage data were obtained from Sakamoto *et al.* (1999) and O'Malley *et al.* (2003).

The extent of genetic association was assessed by determining the degree of: a) interpopulation genetic differentiation based on differences in the allele frequency using the Fisher exact test, with a Markov Chain Monte Carlo approach that provided an estimate of the exact probability (Raymond and Rousset, 1995), b) interpopulation genetic divergence, using the Wright (1965) F_ST_ and Nei (1972) Ds genetic distance indexes, and c) marker-trait associations using the *L*_*D*_ statistic (Choulakian and Mahdi, 2000; Araneda *et al.*, 2009). Further analysis assessed and corrected the population stratification (Pritchard and Rosenberg, 1999; Devlin and Roeder, 1999). The latter analysis served to identify possible spurious associations generated by stratification of the samples and was based on the use of unlinked markers to calculate the lambda factor (λ mean); this factor was subsequently used to correct the statistical significance of the linked marker through the χ^2^ value in a contingency test. All genetic analyses were done using GDA version 1.1 (Lewis and Zaykin, 2001) and TFPGA version 1.3 (Miller, 1997) software packages. Map positions for markers linked to SPT-QTLs were drawn using MapChart software version 2.1 (Voorrips, 2002).

Table 2 summarizes the results of the foregoing analyses. Comparison of Wt-02 with Sh (comparison 1) and Wt-05 with Sh (comparison 2) stocks revealed four linked microsatellites (*OmyFGT12TUF*, *One3ASC*, *One19ASC* and *One112ADFG*) with significant allelic differentiation (p < 0.05) in the Fisher exact test. In addition, two unlinked markers (*OmyFGT15* and *Omy207UoG*) also showed significant allelic differentiation. The linked markers showed higher genetic divergence than those without allelic heterogeneity (comparison 1: Ds = 0.039-0.555 *vs.* 0.022-0.144, F_ST_ = 0.015-0.111 *vs.* 0.012-0.026; comparison 2: Ds = 0.054-0.847 *vs.* 0.070-0.077, F_ST_ = 0,024-0.149 *vs.* 0.025-0.039). Association analysis (*L*_*D*_) was only significant (p < 0.0002) for microsatellites linked to SPT-QTLs, two each in the first (*OmyFGT12TUF* and *One3ASC*) and second (*OmyFGT12* and *One19ASC*) comparisons. These markers had alleles with a significantly higher representation in one of the two populations in each comparison, particularly the 175 bp allele of *OmyFGT12* (Wt-02 = 17.1% *vs.* Sh = 66.7%; Wt-05 = 20% *vs.* Sh = 66.7%), the 203 bp allele of *One3ASC* (Wt-02 = 43.8% *vs.* Sh = 2.1%) and the 127 bp allele of *One19ASC* (Wt-05 = 63% *vs.* Sh = 18%) (Figure 2). Evaluation of comparisons 1 and 2 using the four unlinked markers showed that both comparisons had a significant level of stratification (comparison 1: χ^2^ = 55.346, DF = 25, p < 0.05; comparison 2: χ^2^ = 66.912, DF = 20, p < 0.05). The stratification correction obtained by applying the lambda factor (λ mean, calculated according to Devlin and Roeder (1999)) showed that two linked markers in comparison 1 (*One3ASC* and *One112ADFG*) and one linked marker in comparison 2 (*One19ASC*) were significantly associated with the DARC trait (p < 0.05) (Table 3). In this correction, an unlinked marker with high allelic frequency differentiation (*Omy207UoG*) was excluded to avoid compromising the corrective capacity of the method (Shmulewitz *et al.*, 2004).

**Figure 2:**
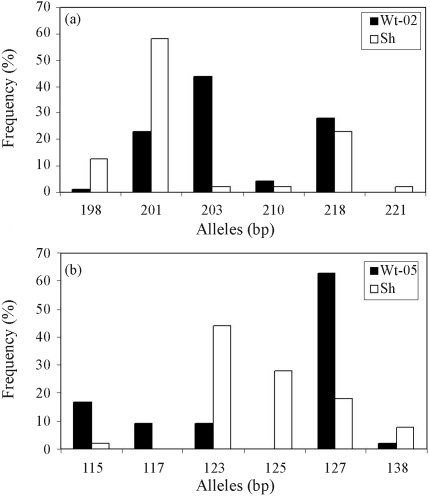
Allelic frequency distributions in the markers *One3ASC* (a) and *One19ASC* (b) linked to spawning time QTLs in Wytheville 02 (Wt-02), Wytheville 05 (Wt-05) and Steelhead (Sh) stocks.

These results support the hypothesis that SPT-QTLs influence the DARC trait in rainbow trout. The QTLs would be those mapped in linkage groups OA-VIII, OA-XIX and OA-XIV of this species, based on information available for the markers linked to these chromosomal regions (Sakamoto *et al.*, 1999; O'Malley *et al.*, 2003). Further studies involving additional markers, as well as case-control groups without selection bias or stratification, are required to assess the association between microsatellites linked to SPT-QTLs and the DARC trait.

Other strategies that could help to clarify the underlying genetics of the DARC trait include a search for candidate genes (Lam, PhD thesis, Universidad de Chile, Santiago de Chile, 2009) and the mapping of QTLs responsible for expression of the trait using backcrosses in experimental populations. Both of these strategies are currently being used in our laboratory and should provide data that will improve our understanding of the genetics of DARC in rainbow trout.

## Figures and Tables

**Table 1:** Description of the nine microsatellite markers analyzed.

Marker	Repeat	Primer sequence	References (GenBank)^*^	Linkage status to SPT-QTLs^#^
*OmyFGT12TUF*	(CA)_36_	F: CAGTGTTGGAACACGTCCTG R: TTGATTCTTGTGATGAAATCGC	1	Linked
*One3ASC*	(GA)_18_	F: TCTCCTTGGTCTCTCTGTCCCTT R: CTATCAGCCAATCGCATCAGGAC	2 (AH003601)	Linked
*One19ASC*	(CA)_33_	F: CTGGAAAGCACAGAGAGAGCCTT R: TCCAACAGTCTAACAGTCTAACCA	2 (U56719)	Linked
*One112ADFG*	(TCTA)_28_	F: GTGACCCAGACTCAGAGGAC R: CACAACCCATCACATGAAAC	3 (AF274528)	Linked
*Ssa103NVH*	(CA)_4_ AA (CA)_14_	F: GCTGTGATTTCTCTCTGC R: AAAGGTGGGTCCAAGGAC	4 (AF256746)	Linked
*OmyFGT14TUF*	(CA)_10_	F: TGAGACTCAACAGTGACCGC R: AGAGGGTTACACATGCACCC	1	Unlinked
*OmyFGT15TUF*	(GT)_8_	F: ATAGTTTCCACTGCCGATGC R: GGTACACACAGCTTGATTGCA	1	Unlinked
*Omy27DU*		F: TTTATGTCATGTCAGCCAGTG R: TTTATGTCATGTCAGCCAGTG	5	Unlinked
*Omy207UoG*	(GT)_31_	F: ACCCTAGTCATTCAGTCAGG R: GATCACTGTGATAGACATCG	6	Unlinked

*1. T Sakamoto, PhD Thesis, Tokyo University of Fisheries, Tokyo, Japan (1996), 2. Scribner *et al.* (1996), 3. Olsen *et al.* (2000), 4. Norwegian Veterinary Hospital, 5. Hologene Inc., Halifax, Nova Scotia, Canada, 6. O'Connell *et al.* (1997). ^#^According to Sakamoto *et al.* (1999), Fishback *et al.* (2000) and O'Malley *et al.* (2003).

**Table 2:** Association analysis between spawning time QTL markers and the double annual reproductive cycle trait in rainbow trout.

Comparison/ marker	Linkage status to SPT-QTLs	Allelic differentiation	Genetic divergence			Marker-trait association	
		p	F_ST_	D_S_		*L*_*D*_	p
1. Wt-02 *vs.* Sh							
*OmyFGT12TUF*	Linked	0.0309*	0.108	0.214		20.959	0.0000**
*One3ASC*	Linked	0.0000*	0.111	0.555		26.525	0.0000**
*One19ASC*	Linked	0.0084	0.026	0.144		8.233	0.0041
*One112ADFG*	Linked	0.0000*	0.015	0.039		7.883	0.0049
*Ssa103NVH*	Linked	0.3225	0.012	0.022		1.229	0.2676
*OmyFGT14TUF*	Unlinked	0.0927	0.005	0.005		5.079	0.0242
*OmyFGT15TUF*	Unlinked	0.1294	0.008	0.018		6.021	0.0141
*Omy27DU*	Unlinked	0.2189	0.013	0.042		2.191	0.1387
*Omy207UoG*	Unlinked	0.0000*	0.041	0.693		3.876	0.0489
2. Wt-05 *vs.* Sh							
*OmyFGT12TUF*	Linked	0.0006*	0.059	0.564		15.496	0.0000**
*One3ASC*	Linked	0.0008*	0.024	0.054		6.915	0.0085
*One19ASC*	Linked	0.0000*	0.149	0.847		21.643	0.0000**
*One112ADFG*	Linked	0.0140	0.025	0.070		9.056	0.0026
*Ssa103NVH*	Linked	0.0650	0.039	0.077		4.323	0.0376
*OmyFGT14TUF*	Unlinked	0.2407	0.015	0.017		1.901	0.1679
*OmyFGT15TUF*	Unlinked	0.0010*	0.026	0.070		8.186	0.0042
*Omy27DU*	Unlinked	0.1743	0.014	0.048		2.216	0.1366
*Omy207UoG*	Unlinked	0.0000*	0.043	0.672		12.716	0.0003

* Significant differences in allelic distribution between broodstock groups after Bonferroni correction with a threshold value of p ≤ 0.05. ** Indicate association with spawning time QTL that is considered significant with a threshold value of p < 0.0002 which corresponds to a chi-squared value > 13.8 with one degree of freedom and equivalent to a LOD score > 3.0.

**Table 3:** Correction for stratification in the association analysis between spawning time QTL markers and the double annual reproductive cycle trait in rainbow trout.

Comparison/ marker	Linkage status to SPT-QTLs	Contingency test		λ correction for the χ^2^ value
		χ^2^	p	
1. Wt-02 *vs.* Sh				λ = 5.801
*OmyFGT12TUF*	Linked	16.275	0.0386*	2.806
*One3ASC*	Linked	41.536	0.0000*	7.160**
*One19ASC*	Linked	14.483	0.0128*	2.497
*One112ADFG*	Linked	27.926	0.0002*	4.814**
*Ssa103NVH*	Linked	1.229	0.2676	0.212
2. Wt-05 *vs.* Sh				λ = 8.545
*OmyFGT12TUF*	Linked	29.877	0.0002*	3.497
*One3ASC*	Linked	15.516	0.0083*	1.816
*One19ASC*	Linked	52.362	0.0000*	6.128**
*One112ADFG*	Linked	15.110	0.0194*	1.768
*Ssa103NVH*	Linked	4.323	0.0376*	0.506

* Significant differences in allelic distribution between broodstock groups with a threshold value of p < 0.05. **Significant differences with a global threshold value of p < 0.05 (χ^2^ > 3.84).
